# Hetero-grafting affects flavonoid biosynthesis in sweet orange 'Newhall' (*Citrus sinensis*) peels: a metabolomics and transcriptomics analysis

**DOI:** 10.3389/fpls.2023.1218426

**Published:** 2023-07-03

**Authors:** Qin Li, Junfei Yao, Wei Zheng, Jialu Wang, Ling Liao, Guochao Sun, Xun Wang, Honghong Deng, Mingfei Zhang, Zhihui Wang, Bo Xiong

**Affiliations:** College of Horticulture, Sichuan Agricultural University, Chengdu, China

**Keywords:** ‘Newhall’ peel, rootstock grafting, flavone, flavonol, metabolomics, transcription factor

## Abstract

Citrus cultivation involves the widespread practice of grafting, which has a significant impact on citrus development and fruit quality and yield. However, understanding the effect of flavonoid compounds after different rootstock grafting have been limited. Flavonoid compounds, found at the highest levels in citrus peels, contribute to improving fruit quality and nutritional value. In this study, scion-rootstock interaction was investigated at various developmental stages when sweet orange 'Newhall' was hetero-grafted with two commonly used rootstocks (*Poncirus trifoliate population, C. junos Siebold ex Tanaka*). Physiological index detection showed a higher concentration of total flavonoid content in peels of sweet orange 'Newhall' grafted on *Poncirus trifoliate population* (ct) than *C. junos Siebold ex Tanaka* (cj). Further metabolomic analysis identified 703 flavonoid compounds, including flavones, flavonols, and flavanones. Out of the 25 flavonoids affected by different rootstock grafting and developmental stages, most were flavones. Transcriptomic analysis identified 8,562 differentially expressed genes (DEGs). Co-expression and Pearson's correlation analysis discovered six hub structure genes and 19 transcription factors (TFs) that affected flavonoid biosynthesis. In addition to increasing the transcript levels of genes that synthesize flavones, flavonols, and flavanones, the scion-rootstock interaction also affected the expression of many TFs. Taken together, our findings suggested that hetero-grafting could promote the accumulation of flavonoid compounds in citrus peels during the development stages. These results offered fresh perspectives on grafting's application usefulness and the enhancement of the accumulation of nutritive flavonoid components by grafting in citrus.

## Introduction

Grown in tropical and subtropical locations, citrus is an essential fruit crop for the entire world. It is widely consumed, and its fruits contain a lot of beneficial flavonoids (Helm and Macdonald, 2015; Nair et al., 2018; Gandhi et al., 2020; Zhao et al., 2021). Citrus may be the ideal choice for studying the flavonoid biosynthesis, which exhibit product- and species-dependence (Jiang et al., 2015; Mulvihill et al., 2016; Mahmoud et al., 2019). Citrus is consumed in numerous various forms, including fresh food, fruit juice, and medicine. And it is a plentiful source of nutrients that have anti-inflammatory, anti-oxidative, immune-modulating, and other disease-preventing properties (Peng et al., 2019; Weiwei et al., 2020). China has seen a huge increase in the production of citrus, with Liangshan's Leibo county one of the biggest producing areas of sweet orange 'Newhall'.

Flavanone glycosides and polymethoxylated flavones (PMFs), which are a series of bioactive substances, are typically accumulated in citrus species (Karn et al., 2021; Zhao et al., 2021). The most prevalent flavonoids are flavonols, which are found naturally in plant vacuoles as glycoside derivatives (Xing et al., 2021). Most citrus species are incapable of producing anthocyanins, but they are excellent study materials for flavanones and flavones in fruit (Fabroni et al., 2016; Briskey et al., 2022). Plant growth, adaptability, signal transduction, and resistance to biotic and abiotic stressors are all impacted by flavonoids (Li and Schluesener, 2017; Li et al., 2017). Glycosylation changes flavonoids, resulting in a variety of chemical forms and biological functions (Yang et al., 2016; Ito et al., 2017; Yu et al., 2022). Many TFs influenced flavonoid biosynthesis pathway, including R2R3-MYBs and AP2/ERF-ERFs proteins (Li et al., 2020; Zhao et al., 2021; Fan et al., 2022; Zhang et al., 2022a; Meng et al., 2023; Ni et al., 2023). Different TFs governed the formation of various flavonoids (Liu et al., 2016; Matus et al., 2017; Zhao et al., 2021).

Various factors regulated the accumulation of secondary metabolites. The biosynthesis of secondary metabolites has been shown to be affected by grafting, and as a result, grafted plants metabolite compositions differed from non-grafted plants (Habran et al., 2016; Kyriacou et al., 2017; Xu et al., 2017; Deng et al., 2018; Dong et al., 2022). When compared to ungrafted grapevines, grafted grapes were shown to have higher amounts of total proanthocyanidins (PAs), flavonols, and other PA components in the fruit peels (Zhang et al., 2022b). Similar to this, grafted watermelons were reported to accumulate more lycopene (Proietti et al., 2008). There were, however, few research on the molecular mechanisms that control scion-rootstock interactions. Therefore, examining how citrus development and the accumulation of flavonoid molecules were affected by rootstocks would provide a critical foundation for enhancing flavonoids.

Studies of rootstock-mediated effects in regulating gene expression and secondary metabolites have been conducted using multi-omics methodologies as transcriptomics, proteomics, and metabolomics (Morrow et al., 2001; Lu et al., 2014; Matus, 2016). The accumulation of organic acids, sugars, and phenols in grapes grafted by various rootstocks was considerably different according to transcriptome and metabolome data (Chitarra et al., 2017; Zhang et al., 2022a; Zhang et al., 2022b; Zhang et al., 2022c). However, limited information is available on the impact of virous rootstocks grafting on dynamic metabolomic and transcriptomic alterations in citrus throughout distinct developmental periods.

The purpose of this study was to investigate flavonoid accumulation and gene expression in citrus peels throughout growth of two scion-rootstock combinations. Due to their better tolerance to abiotic and biotic stressors, the widely used *Poncirus t*rifoliate *population* and *C. junos Siebold ex Tanaka* rootstocks were employed for citrus grafting globally (Peng et al., 2020; Huang et al., 2021). A metabolomic approach was used to quantify flavonoid compounds at various developmental periods, and a transcriptome technique was applied to examine changes in gene expression. These studies provided important new insights into how rootstock grafting regulated the accumulation of flavonoids in sweet orange 'Newhall' peel, and might lead to improvement of methods for enhancing its nutritional value.

## Materials and methods

### Plants and sample preparation

**Figure 1:**
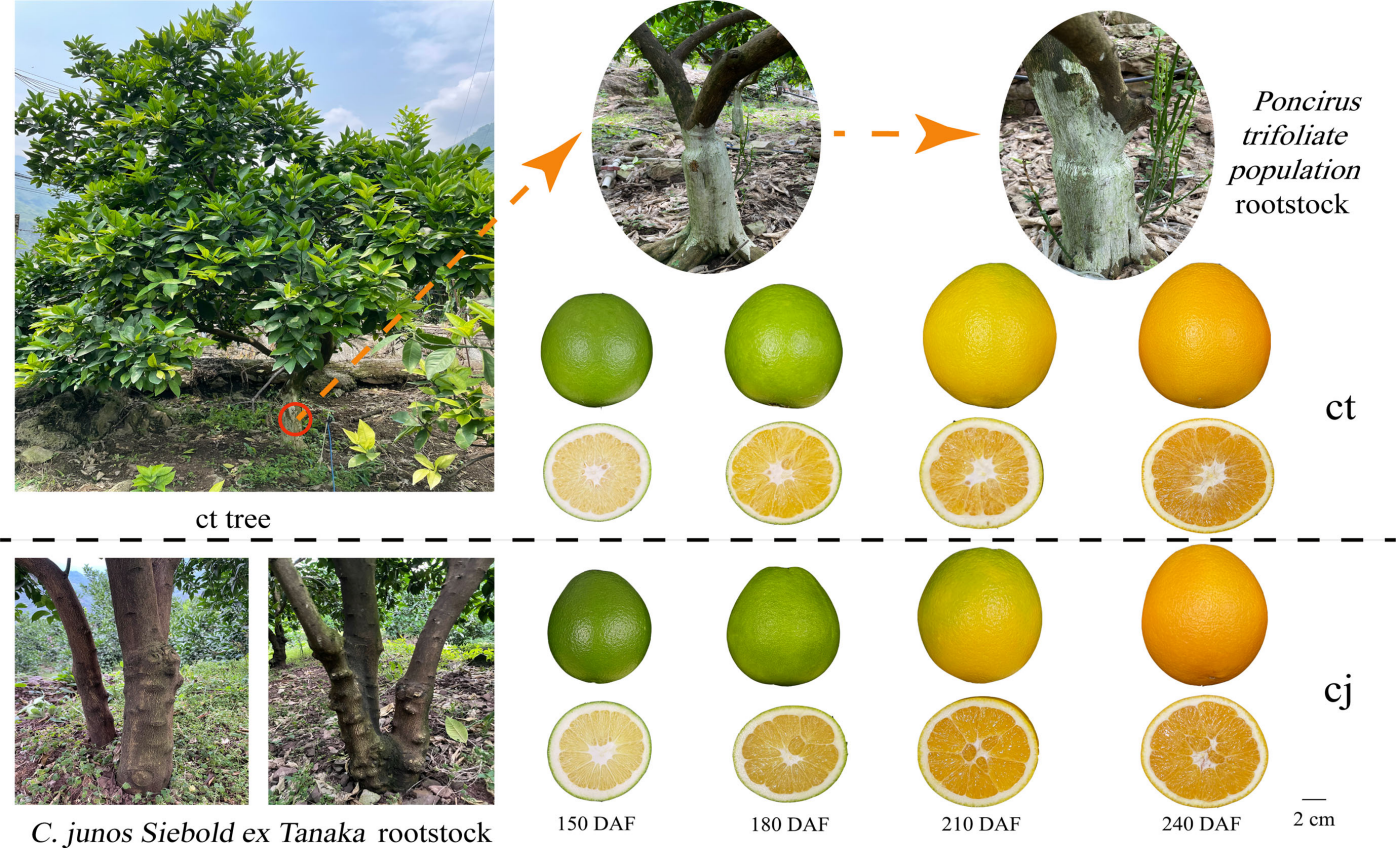
The rootstocks from *Poncirus t*rifoliate *population* (ct) and *C. junos Siebold ex Tanaka* (cj) and collected fruit at fruit expansion period (150 days after flowering), late fruit expansion period (180 days after flowering), fruit turning period (210 days after flowering) and fruit ripening period (240 days after flowering).

The experiments were carried out in sweet orange 'Newhall' orchard located in Guan village, LeiBo county, LiangShan prefecture, Sichuan province, China. This region was situated 1000 meters below sea level in the Jinsha River valley area and experienced a tropical monsoon climate with more than 1000 hours of sunshine annually. The soil pH was 8.25. Sweet orange 'Newhall' (*Citrus sinensis*) was used as scion, with rootstock from a 13-year-old of *Poncirus t*rifoliate *population* (ct) and *C. junos Siebold ex Tanaka* (cj). The field management was consistent. Citrus samples from each scion-rootstock combination were collected at four stages, including fruit expansion period (150 days after flowering), late fruit expansion period (180 days after flowering), fruit turning period (210 days after flowering), and fruit ripening period (240 days after flowering) (**Figure 1**). At each sampling date, three independent biological replicates were randomly obtained, and six trees were used per biological replicate. Fruits of the same size without pests and diseases were collected from five directions, outside the crown (east, south, west, north, and middle). Each tree collected 5 fruits. The fruits were immediately brought back to the lab for processing, washed in deionized water, and separated into peels and pulps. Part of the peels were frozen and stored at -40°C for total flavonoid content detection, while another portion of the peels were frozen quickly into liquid nitrogen, packed into 10 mL sterile tubes without enzymes, and placed in a -80°C cryogenic refrigerator for metabolomic and transcriptomic analyses.

### Measurement of total flavonoid content

Total flavonoid content of sweet orange 'Newhall' peels was measured using a modified version (Wang et al., 2007; Yu et al., 2022). Weighed 0.5 g of fresh peel and ground it into powder by machine, then dissolved in 10 mL of 70% ethanol (1:20, w/v). Ultrasonic treatment was performed at 55°C for 40 minutes, followed by filtration. 1 mL of the extraction solution was then added sequentially with 0.5 mL of 5% NaNO_2_ solution, shaken well, and left for 5 minutes. Subsequently, 0.5 mL of 10% Al (NO_3_)_3_ was added, mixed, and left for 6 minutes. Finally, 5 mL of 1 mol/L NaOH was added, pure water was added to 10 mL, and the mixture was shaken and left for 10 minutes. The absorbance value was assessed at 510 nm by UV-Vis spectrophotometer after the reaction. Rutin (purity ≥ 98%, Source leaf Shanghai Biological Technology co., LTD) was selected as standard.

### Metabolites extraction and analysis

Metabolite detection was conducted by Wuhan Meteware Biotechnology Co., Ltd. Flavonoid metabolites were identified and quantified using ultra-performance liquid chromatography and tandem mass spectrometry (UPLC-MS/MS) analysis. The 18 samples were first vacuum freeze-dried using Scientz-100F and subsequently ground to powder using a grinder (MM400, Retsch) at 30Hz for 1.5 minutes. 50 mg of powder was weighed and 1200 µL of 70% methanol was added, followed by vortexing for six times. After centrifuging the samples (12 000 rpm, 3 min, 4°C), the supernatant liquid was extracted and filtered using a microporous filtration membrane (0.22 µm) before being stored in a sample vial for UPLC-MS/MS analysis. The column used Agilent SB-C18 (1.8 µm, 2.1 mm × 100 mm). The mobile phases A and B were ultra-pure water added with 0.1% formic acid and acetonitrile added with 0.1% formic acid, respectively. The solvent gradient used was 5% B at 0 min, 5-95% B in 9.0 min, 95% B in 10.0-11.1 min, 95-5% B in 11.1-14.0 min, and 5% B at 14 min. The column temperature was 40°C, the flow rate was 0.35 mL / min, and the sample size was 2 µL.

The mass spectrum conditions mainly included the temperature of electrospray ionization (ESI) was 500°C, and the ion spray voltage was 5500V (positive ion mode) / -4500V (negative ion mode). The ion source gases GSI, GSII, and CUR were set at 50, 60, and 25 psi, respectively. The collision-induced ionization parameter was set to high. The QQQ scan used MRM mode and the collision gas (nitrogen) was set to medium. The DP and CE of each MRM ion pair were completed by further optimization of declustering potential (DP) and collision energy (CE). A specific set of MRM ion pairs was monitored at each period based on the metabolites eluted during each period. The quantization of metabolites was achieved by multi-reaction monitoring mode (MRM) and triple quadrupole mass spectrometry. Mass spectrum data was processed using Analyst v 1.6.3 software.

### Whole-transcriptome sequencing and transcriptomic analysis

The 18 samples were delivered to Metware Biotechnology Co., Ltd (Wuhan, China) for RNA sequencing. Total RNA was extracted from ct and cj peels using RNAprep Pure Plant Plus Kit (Polysaccharides&Polyphenolics-rich) (Tiangen Biotech Co. Ltd, China). The acquired RNA was quantified by a Nanodrop spectrophotometer and tested by gel electrophoresis. A total amount of l μg RNAper sample was used as input material for the RNA sample preparations. Sequencing libraries were generated using NEBNextRUltraTMRNA Library Prep Kit for lluminaR (NEB,USA) following manufacturer’s recommendations. After the library was constructed, Qubit2.0 Fluorometer was used for preliminary quantification and the library was diluted to 1.5 ng/μL, followed by Agilent 2100 bioanalyzer for Insert Size detection. After Insert Size met expectations, the library effective concentration was set to 2nM.

PCR amplification and sequencing of 18 cDNA libraries were carried out using the Illumina novaseq 6000 platform. The clustering of the index-coded samples was performed on a cBot Cluster Generation System using TruSeq PE Cluster Kit v3-cBot-HS (Illumia) according to the manufacturer’s instructions. After cluster generation, the library preparations were sequenced on an Illumina platform and 150 bp paired-end reads were generated. We used fastp to apply strict quality control to the data to ensure clean reads before conducting data analysis. The sequences obtained by RNA-seq were mapped to the reference genome sequence of *C. sinensis* v3.0 (http://www.hzau.edu.cn), and then StringTie assembled it. The fragments per kilobase per million (FPKM) value was used to quantify and normalize gene abundance. With a significance criterion of p-value < 0.05 and fold change (FC) > 2 or < 0.5, DEGs were identified using the DESeq2 software.

### Co-expression network and quantitative real-time PCR (qRT-PCR) analysis

Using the free online data analysis platform Metware Cloud (https://cloud.metware.cn), co-expression network was constructed to search for TFs that were co-expressed with structural genes. Actin was used as endogenous control, and the expression levels of 25 genes were detected by qRT-PCR analysis to validate the transcriptome profiling (**Table S1**). Total RNA was extracted by Trizol with slight improvement. The reagents were made from Super TRIgent extract, 75% ethanol prepared with DEPC water, trichloromethane, isopropyl alcohol and DEPC water. RNA samples with OD260/OD280 greater than 1.8 and less than 2.1 were selected for reverse transcription. Reverse transcription was performed using a kit (Mei5 Bioservices Co. Ltd, China). The template RNA was 0.2 μg in the reverse transcription system. The qRT-PCR was performed in a 12.5 μL reaction system (1 μL cDNA, 6.25 μL 2×M5SYBR, 1 μL upstream and downstream primers, and 4.25 μL DEPC water). qRT-PCR used a two-part amplification method: 95°C, 2 min; 95°C, 5 seconds, TM, 40 seconds, 39 cycles; 95°C, 10 seconds, 65°C, 1 seconds, 95°C, 5 seconds. qRT-PCR was monitored with a CFX96 Real-Time PCR Detection System (Hercules, CA, US) and the 2^−△△Ct^ method to calculate cycle threshold values and obtain melting curves.

### Statistical analysis

The data significance analysis was calculated using IBM SPSS Statistics 24.0. Duncan’s test and one-way ANOVA were used to compare the differences between ct and cj, and the difference was deemeded as significant when p ≤ 0.05. Pearson correlation analysis was carried out using the Metware cloud platform (https://cloud.metware.cn). Related parameters used the platform default values. The 2^−△△Ct^ method was used to analyze the data.

## Results

### Suitable rootstock grafting promotes the accumulation of total flavonoid content in citrus peels

**Figure 2:**
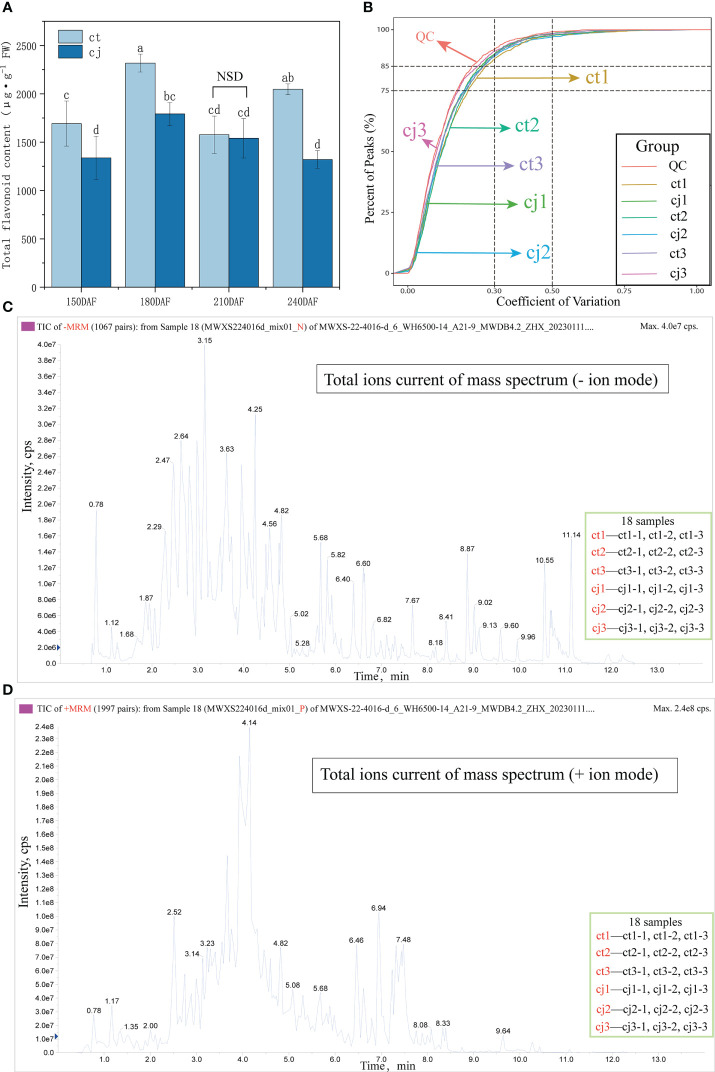
Qualitative and quantitative analyses of flavonoid metabolites in ct and cj peels. **(A)** Total flavonoid content in ct and cj peels. Values in each column with different letters indicated significantly different at P<0.05. ‘NSD’ represented no significant difference. **(B)** CV value distribution diagram. The x-coordinate represented the CV value, and the y-coordinate represented the proportion of the number of substances less than the corresponding CV value to the total number of substances. Different colors represented different groups of samples. The proportion of QC samples with CV value less than 0.5 was higher than 85%, indicating that the experimental data was stable. The proportion of QC samples with CV value less than 0.3 was higher than 75%, indicating that the experimental data was quite stable. **(C, D)** Total ions current diagram of sample quality spectrum analysis. The x-coordinate was the retention time (min) of the metabolite detection and the y-coordinate was the ion current intensity (cps) of a metabolite ion detection. **(C)** represented the negative (-) ion mode and **(D)** indicated the positive (+) ion mode.

Samples of ct and cj were collected at four stages of development, 150DAF, 180DAF, 210DAF and 240DAF (**Figure 1**). Total flavonoid content increased from 150DAF (1692.42 μg·g^-1^, 1337.38 μg·g^-1^) to 180DAF (2317.67 μg·g^-1^, 1791.67 μg·g^-1^) in ct and cj, reaching its peak at 180DAF (**Figure 2A**). However, there was no appreciable distinction in total flavonoid content between ct and cj at 210DAF. At 150DAF, 180DAF and 240DAF, total flavonoid content was significantly higher in ct than in cj. As a result, samples of 150DAF (ct1, cj1), 180DAF (ct2, cj2) and 240DAF (ct3, cj3) were chosen for subsequent experiments. A UPLC-MS/MS was used to study the differential flavonoids in 18 selected samples from three developmental stages, in which a total of 703 flavonoids were detected (**Table S2**). Coefficient of variation (CV) value could reflect the degree of dispersion of data. When the proportion of substances with CV value less than 0.3 in QC (quality control) samples was higher than 75%, it indicated that the experimental data was very stable. In this study, CV values less than 0.3 of QC samples accounted for more than 85%, indicating that our experimental data were relatively stable and available (**Figure 2B**). The peaks of positive and negative ion patterns in the total ion current pattern were smooth, which further indicated the availability of metabolome data (**Figures 2C, D**).

**Figure 3:**
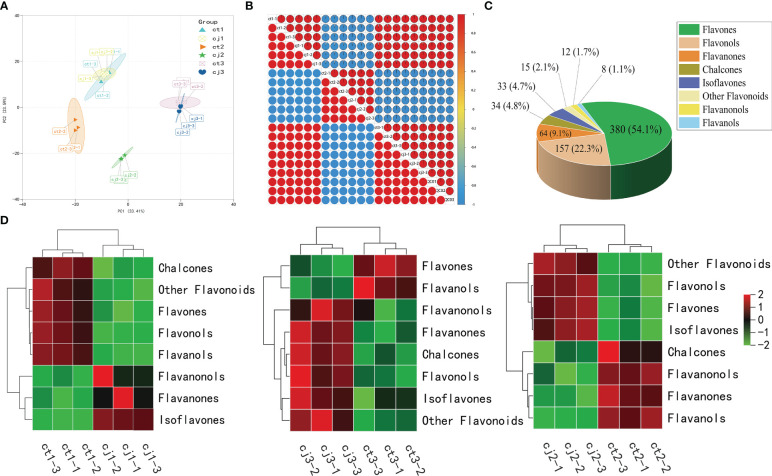
Flavonoid metabolome profiling of ct and cj peels. **(A)** Principal component analysis (PCA) of flavonoids in 18 different samples. **(B)** Intra-group and intergroup correlation of 18 samples at different stages. **(C)** Classification and proportion of 703 flavonoids in ct and cj peels. **(D)** Heatmap of 8 classes flavonoid in ct and cj peels over three developmental periods, respectively. Red represents the high flavonoid content. Green represents the low flavonoid content.

Data availability was verified by PCA and correlation analysis (**Figures 3A, B**). PCA indicated a high correlation between repetitions, while Pearson’s correlation coefficient demonstrated that the obtained data were reliable and reproducible. In addition, ct2 and cj2 were significantly different, which was consistent with the trend in total flavonoid content change, signifying the accuracy of total flavonoid content determination (**Figure 2A**). To further analyze the compounds that contribute the most to the total flavonoid content, we classified 703 flavonoids into eight classes, with flavones accounting for the highest proportion of flavonoids (380, 54.1%), followed by flavonols (157, 22.3%) and flavanones (64, 9.1%) (**Figure 3C**). Heatmap analysis revealed that chalcones, flavones, flavanols, flavonols, and other flavonoids made significant contributions to ct1 (**Figure 3D**). Chalcones, flavanonols, flavanones, and flavanols resulted in higher total flavonoid content in ct2 than cj2, while only flavones and flavanols made major contributions in ct3. The analysis showed that flavones might be the most important flavonoid class responsible for the difference between ct and cj peels in total flavonoid content.

### Metabolomic studies revealed differential accumulation of flavonoids (DAFlv) between ct and cj peels

**Figure 4:**
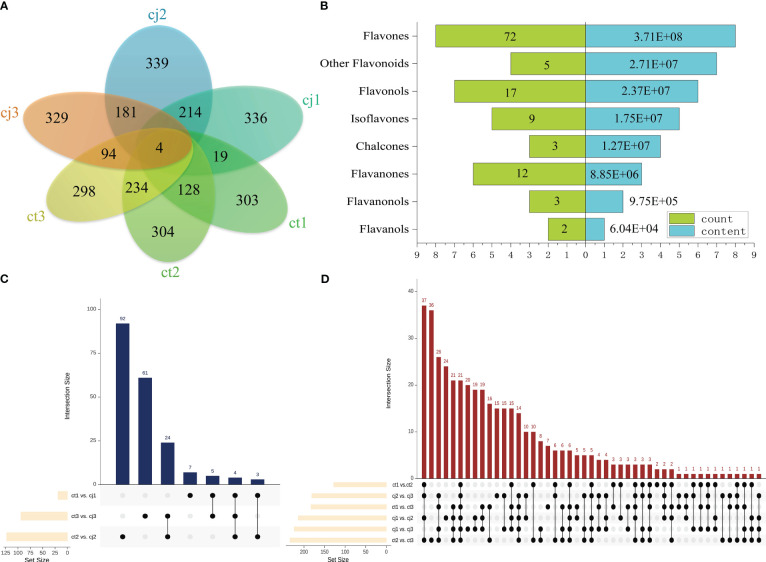
Differential analysis of flavonoids in ct and cj peels. **(A)** Venn diagram of ct1, cj1, ct2, cj2, ct3, and cj3. **(B)** Inverted pyramid diagram of 123 flavonoids in ct2 vs cj2. The left side represents flavonoid counts, and the right side represents flavonoid content. **(C, D)** Upset diagram. The column chart shows the numbers of nine groups.

DAFlv were defined with FC values more than 2 or less than 0.05. A Venn diagram was used to illustrate the distribution of DAFlv among six groups (**Figure 4A**). A total of 303, 304, 298, 336, 339, and 329 DAFlv were identified in ct1, ct2, ct3, cj1, cj2, and cj3, respectively. In particular, 123 DAFlv overlapped in ct2 vs cj2, which had the largest number of DAFlv compared to ct1 vs cj1 (19) and ct3 vs cj3 (94). An inverted pyramid analysis was performed on these 123 DAFlv (**Figure 4B**; **Table S3**), with the largest numbers of flavones (72), flavonols (17), and flavanones (12). However, the number of DAFlv did not correlate with total flavonoid content in eight classes. Flavones had the highest total content at 3.71E+08, followed by other flavonoids and flavonols at 2.71E+07 and 2.37E+07, respectively, while flavanones contributed minimally.

A total of 92, 61, 24, 7, 5, and 3 flavonoids were identified in ct2 vs cj2, ct3 vs cj3, ct2 vs cj2 and ct3 vs cj3, ct1 vs cj1, ct1 vs cj1 and ct3 vs cj3, and ct1 vs cj1 and ct2 vs cj2, respectively (**Figure 4C**). Four flavonoids (3,4’-Dihydroxyflavone, Chrysoeriol-7-O-(6’’-feruloyl)glucoside, Cirsimaritin 5-[6’’-(3-Hydroxy-3-Methylglutaryl)Glucoside], and Tricin-7-O-(2’’-Sinapoyl)glucuronide) belonging to flavones were present in three comparison groups, indicating that they were affected by different rootstock grafting. In addition, pairwise comparisons were conducted between different developmental stages (**Figure 4D**). A total of 21 flavonoids were shared among six comparisons, including 15 flavones, 2 flavonols, 2 other flavonoids, 1 flavanone, and 1 isoflavone, indicating that they were significantly affected by different developmental periods. These 25 flavonoids were used for subsequent analysis.

### Gene expression profiles in ct and cj peels during three periods

**Figure 5:**
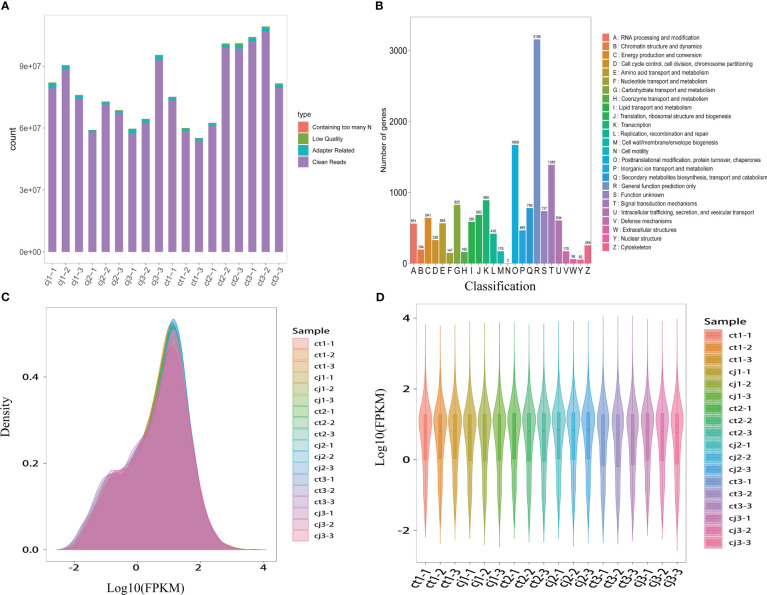
Transcriptome sequencing and functional analysis of DEGs in ct and cj peels. **(A)** Raw sequencing data composition. Containing too many N represents the proportion of reads containing N bases, Low Quality represents the proportion of reads with low sequencing quality, and Adapter Rrelated indicates the percentage of reads with connectors. **(B)** KOG classification bar chart. The X-axis represents the functional classification of KOG ID and Y-axis represents the number of differential genes included. **(C)** Expression density distribution diagram. The x-coordinate represents the pair value of the corresponding sample FPKM, and the y-coordinate represents the probability density. **(D)** Violin map of gene expression. Different colors represent different samples. The width of each violin reflects the number of genes at that level of expression.

18 samples were submitted to RNA-seq analysis to study the probable molecular mechanism of flavonoid biosynthesis in ct and cj peels at various stages. All transcriptional information was stored in the NCBI Sequence Read Archive (BioProject: PRJNA953932). High-throughput sequencing yielded 57,460,244 to 106,692,752 clean reads, generating over 200 Gb of clean base with Q30 percentages (percentage of sequences with sequencing error rates < 0.1%) and GC percentages ranging from 91.41% to 94.04% and 44.27% to 44.66%, respectively (**Figure 5A**; **Table S4**). Using the KEGG, NR, Swissprot, Tremble, KOG, GO, and Pfam databases, predicted protein sequences were annotated. As a result, 25,483 unigenes had at least one database annotation (**Table S5**). All unigenes in ct and cj peels were divided into 25 categories based on gene function, and 683, 890, 780, and 1,388 unigenes were significantly enriched in “Translation, ribosomal structure, and biogenesis”, “Transcription”, “Secondary metabolites biosynthesis, transport, and catabolism”, and “Signal transduction mechanisms” (**Figure 5B**). Gene density mapping illustrated the changes in gene abundance and clearly reflected the concentration of gene expression (**Figure 5C**). Violin plots showed the distribution and probability density of multiple omics data, demonstrating good repeatability within each sample group and considerable separation in different groups, indicating the high quality of RNA-seq data.

### Identification and functional enrichment analysis of DEGs

**Figure 6:**
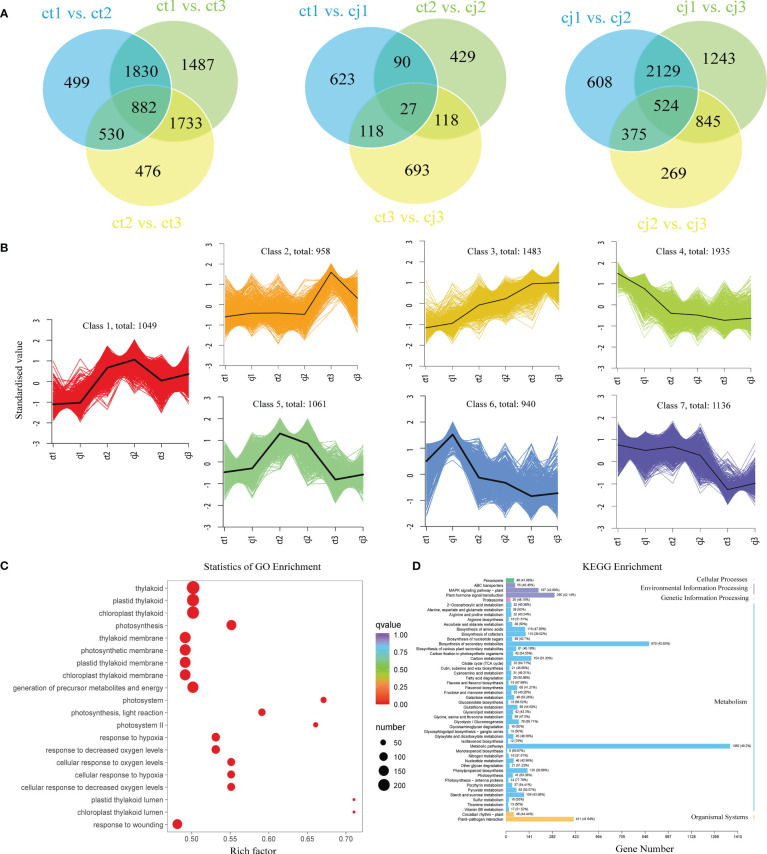
Analysis of DEGs enrichment pathways and expression patterns. **(A)** Venn diagrams of DEGs in ct and cj peels. **(B)** Kmeans diagrams of DEGs. The abscissa represents the sample and ordinate represents the standardised value. **(C)** GO enrichment circle diagram of DEGs in ct and cj, respectively. Bubble size indicates the number of DEGs. Bubble color represents *P*-values. **(D)** KEGG enrichment column diagrams of DEGs in ct and cj, respectively.

Venn diagrams were used at three developmental stages to identify DEGs (**Figure 6A**). For each pairwise comparison, the DEGs were screened using the expression level criterion | log2(fold change) | > 1 and FDR < 0.05. According to the findings, ct1 to ct3 shared 882 DEGs while cj1 to cj3 shared 524 DEGs, indicating that these DEGs were to blame for the increased total flavonoid concent in ct peels. The FPKM of the union of all DEGs was first standardised using the scale function of the R programming language, followed by K-means clustering analysis, to examine gene expression patterns under various rootstock grafting circumstances (**Figure 6B**). Equivalent genes showed comparable alterations and functionality. All DEGs were classified into 7 categories, with class 2, 4, 5, 6, and 7 potentially containing genes influencing flavonoid biosynthesis between ct and cj peels. The expression trends of these five classes were consistent with total flavonoid content in ct and cj peels.

Various genes involved in biological processes (BP), cellular components (CC), and molecular functions (MF) were discovered by gene ontology (GO) enrichment analysis of DEGs (**Figures 6C**, **S1**–**S4**). BP enriched over 1000 DEGs, including signaling and metabolic pathways. The GO enrichment analysis of DEGs revealed that flavonoid transmembrane transport was closely related to biological activities. In addition, many DEGs were enriched in MF associated with flavonoid accumulation, including transcription regulator activity, transporter activity, molecular function regulator, and translation regulator activity (**Figures S1**, **S2**). Additional information on the biological roles of the identified DEGs was revealed by the findings of the Kyoto encyclopedia of genes and genomes (KEGG). ‘Plant hormone signal transduction’, ‘flavone and flavonol biosynthesis’, ‘flavonoid biosynthesis’, ‘fructose and mannose metabolism’, ‘glycolysis/gluconeogenesis’, ‘isoflavonoid biosynthesis’, ‘phenylpropanoid biosynthesis’, and ‘starch and sucrose metabolism’ were all pathways in which these DEGs were enriched (**Figure 6D**).

### Rootstock grafting promotes transcription level of genes related to flavonoid biosynthesis pathway

**Figure 7:**
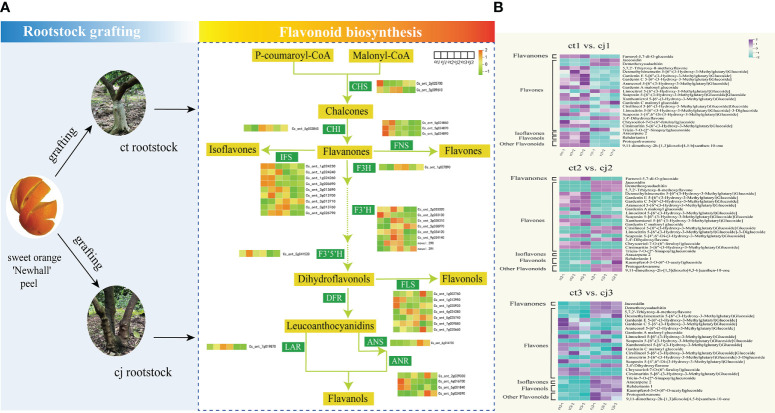
Flavonoid biosynthesis pathway in ct and cj peels. **(A)** Relationship between hub structural genes and metabolite synthesis in flavonoid biosynthesis pathway. **(B)** Content distribution of 25 flavonoids in ct and cj at three stages.

Based on KEGG pathway enrichment and GO function analyses, 38 unigenes encoding enzymes involved in flavonoid biosynthesis were identified in this study. These included 2 *CHS* genes, 1 *CHI* gene, 3 *FNS* genes, 9 *IFS* genes, 1 *F3H* gene, 8 *F3’H* genes, 1 *F3’5’H* gene, 7 *FLS* genes, 1 *LAR* gene, 1 *ANS* gene, and 4 *ANR* genes (**Figure 7A**; **Table S5**). We compared the relative content of 25 flavonoids in pairs between ct and cj (**Figure 7B**; **Table S6**). When ct1 and cj1 were compared, 6 flavonoids increased and 17 reduced, but when ct2 and cj2 were compared, 13 and 12 flavonoids upregulated and downregulated, respectively. Furthermore, 14 flavonoids increased, and nine flavonoids decreased between ct3 and cj3. The findings revealed that the content of flavonoids exhibited different trends in different comparisons.

**Figure 8:**
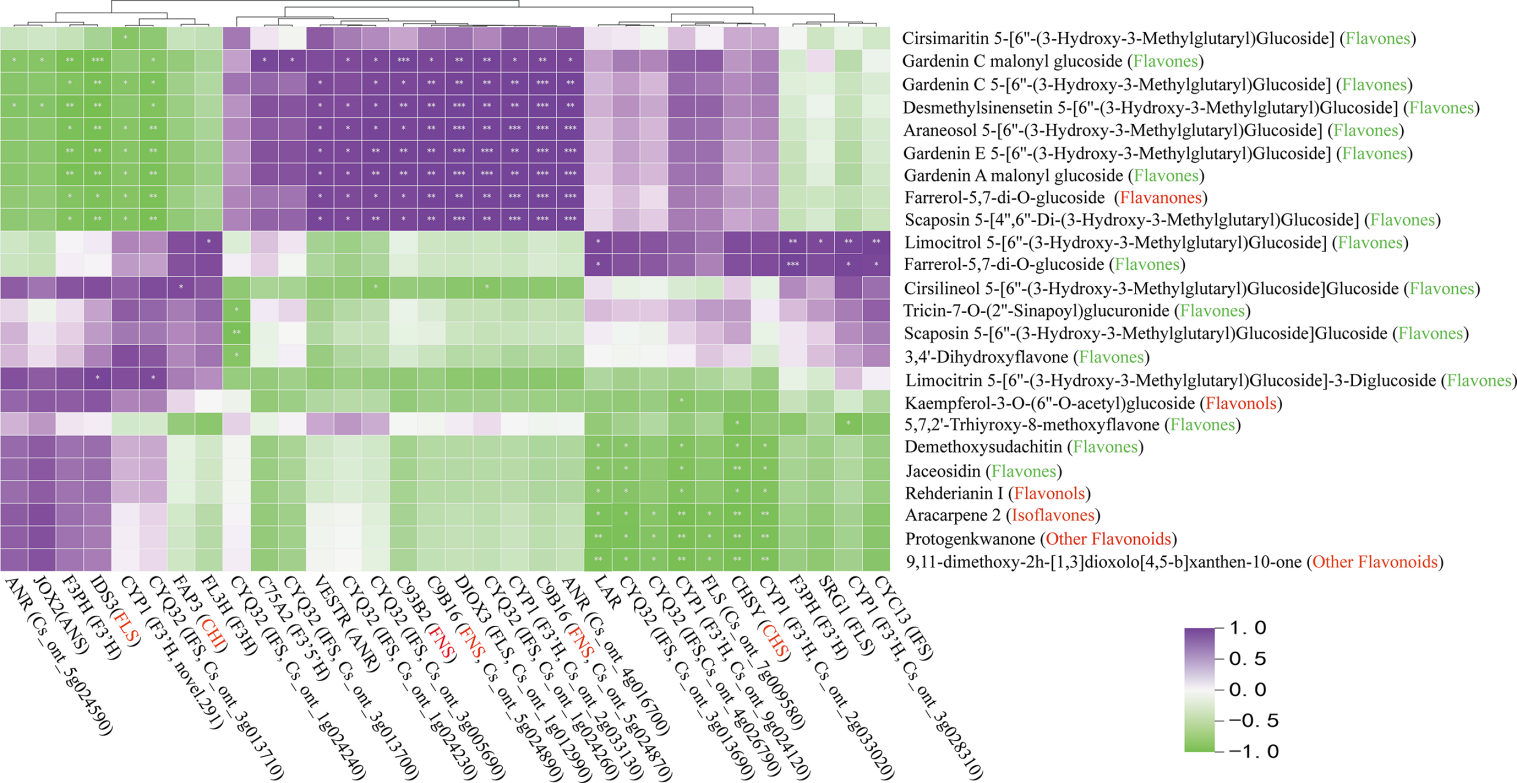
Pearson correlation analysis between flavonoid structural genes and metabolites in flavonoid synthesis pathway. The red text on the horizontal axis represents significantly associated structural genes. The red and green text in vertical brackets represents flavonoid classification, with significant ones marked with * (p < 0.05), **P<0.01, ***P<0.001.

**Figure 9:**
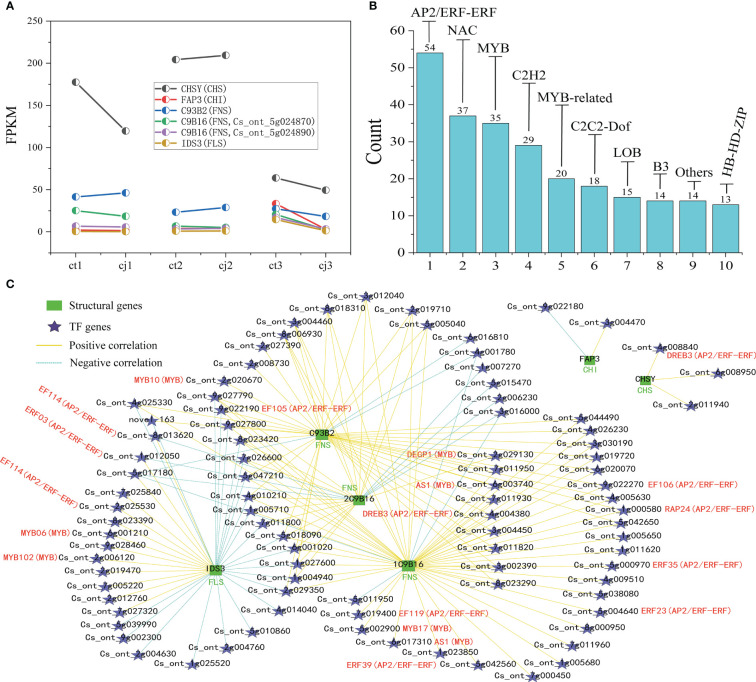
Screening of TFs and co-expression network analysis of DEGs. **(A)** Two-point diagram of Fragments Per Kilobase of exon model per million mapped fragments (FPKM) in 6 hub structural genes. **(B)** Bar chart of the top 10 TF families. **(C)** Co-expression network of six synthetic genes with the top 10 TF families. The green bar graph represents structural genes, five-pointed stars represent TFs, yellow line represents high positive correlation, and azure dotted line represents negative correlation.

The expression levels of the 38 candidate genes and flavonoid content were performed in the biosynthesis pathway using Pearson’s correlation analysis (**Figure 8**). The results indicated that one unigene for *CHS* (*CHSY*), one unigene for *CHI* (*FAP3*), three unigenes for *FNS* (*C93B2*, 2*C9B16*), and one unigene for *FLS* (*IDS3*) were significantly correlated with one flavanone, one flavonol, and most flavones. A two-point diagram revealed that the *CHS* gene (Cs_ont_3g009610) showed the same trend as the *FLS* (Cs_ont_5g025740) gene, while the *CHI* (Cs_ont_5g033840) gene exhibited a similar trend to two *FNS* (Cs_ont_5g024870, Cs_ont_5g024890) genes (**Figure 9A**). The results showed that 6 unigenes were crucial in regulating flavonoid biosynthesis, especially in flavones biosynthesis. These findings provided important insights into the regulatory mechanisms of flavonoid biosynthesis and the identification of key genes for future research.

### Conjoint analysis of transcriptome and metabolome data

To investigate the TFs regulating the differential accumulation of six hub structural genes in ct and cj peels, we further analyzed the transcripts of TF encoding genes. A total of 1,887 TFs were identified, of which 643 were expressed differently (**Table S7**). Among these, one TF was annotated to the flavonoid biosynthesis pathway, while 447 were unannotated using KEGG, NR, Swissprot, Tremble, KOG, GO, and Pfam databases, suggesting that these 448 TFs might regulate DAFs. The top 10 TF families included 249 differentially expressed TFs, including *AP2/ERF-ERF*, *NAC*, *MYB*, *C2H2*, *MYB-related*, *C2C2-Dof*, *LOB*, *B3*, *HB-HD-ZIP*, and Others (**Figure 9B**; **Table S8**).

**Figure 10:**
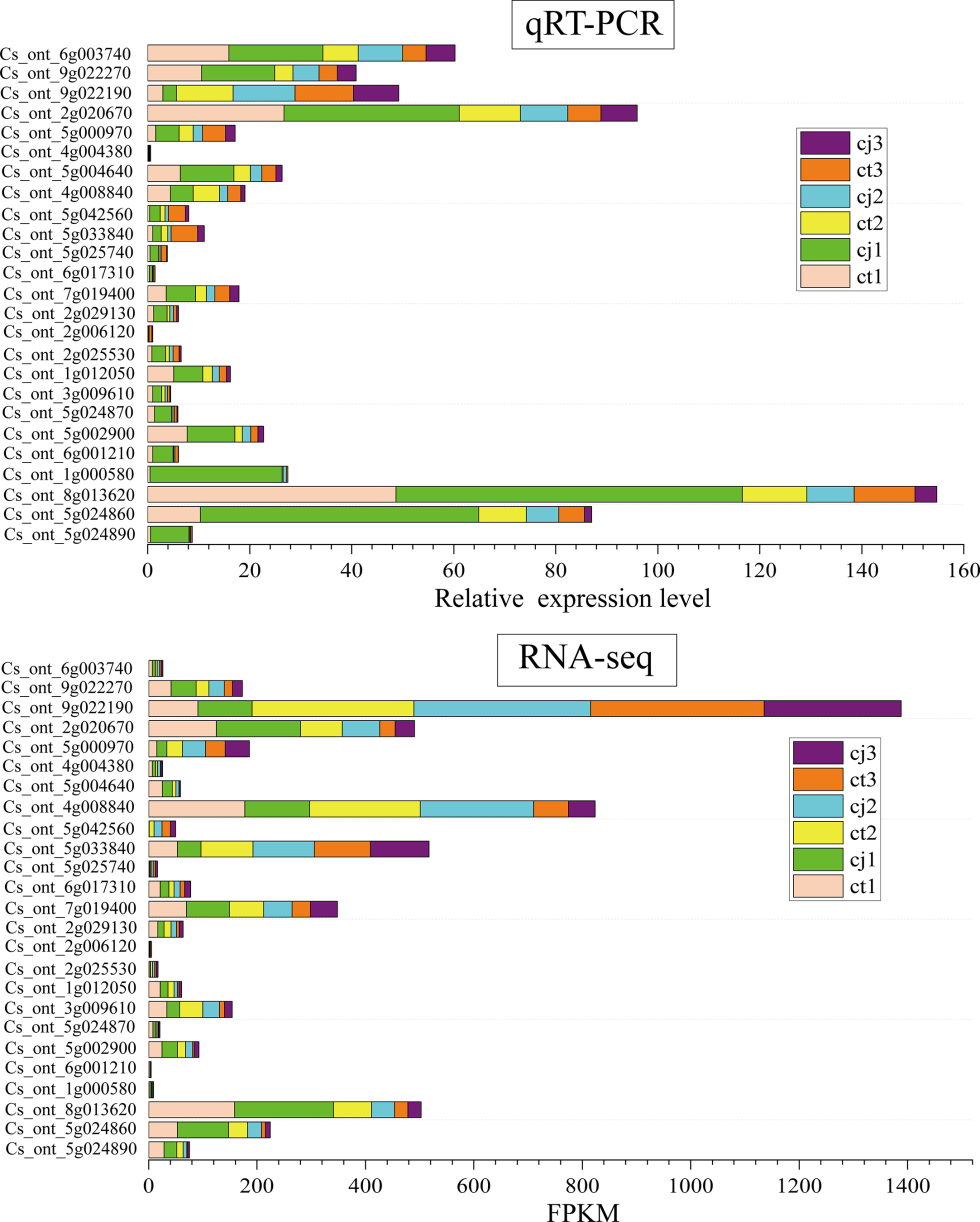
The relative expression levels of qRT-PCR in ct and cj peels at three different periods.The stacked bar plot at the top represents the relative expression levels of qRT-PCR. The stacked bar plot below represents the FPKM values of RNA-seq.

The regulatory network of flavonoid biosynthesis between the six hub structural genes and 249 differentially expressed TFs was evaluated in ct and cj peels (**Figure 9C**). In the regulatory network, the *CHS* gene was positively correlated with three TFs, including 1 *AP2/ERF-ERF* TF (CsDREB3). Five TFs (2*CsEF114*, *CsERF03*, *CsMYB06* and *CsMYB102*) were positively correlated with the *FLS* structural gene, while one TF had a negative correlation. In addition, 11 TFs (*CsEF105*, *2CsAS1*, *CsDREB3*, *CsEF119*, *CsMYB17*, *CsERF39*, *CsEF106*, *CsRAP24*, *CsERF35* and *CsERF23*) were positively correlated with *C9B16* (*FNS*, Cs_ont_5g024870), and five TFs (CsMYB10, CsAS1, CsDREB3, CsEF106 and CsRAP24) were positively correlated with *C93B2* (*FNS*). Two TFs were negatively correlated with *C9B16* (FNS, Cs_ont_5g024890), but only one TF was positively correlated with it. These results indicated that the above 19 TFs could regulate the expression of related structural genes, leading to differential flavonoid accumulation. Finally, we performed qRT-PCR for the above six structural genes and 19 TFs, and the results showed the dependability and precision of transcriptome data (**Figure 10**).

## Discussion

Rootstock grafting is one of the most common activities in citrus production, which directly affects citrus fruit quality and flavor. In grapes, grafting has been shown to affect flavonoid biosynthesis (Zhang et al., 2022b; Zhang et al., 2022c). However, the molecular mechanism of flavonoid biosynthesis by grafting different rootstocks in citrus remains unclear. In this study, metabolomics and transcriptomics were combined to analyze flavonoid biosynthetic pathway in sweet orange ‘Newhall’ peels after rootstock grafting. Furthermore, the structural genes and TFs affecting flavonoid biosynthesis were screened by combining DAFs and DEGs to analyze the potential molecular mechanism of flavonoid biosynthesis in sweet orange ‘Newhall’ peels.

In our experiment, we found that flavones were the most diverse and abundant, followed by flavanols and flavanones (**Figure 3C**). Previous studies have also found flavones were the most abundant class of flavonoids in citrus peels (Wang et al., 2017c; Zhao et al., 2021). However, there were more flavanols than flavanones, suggesting that flavones and flavanols were the two most abundant flavonoids in citrus peel, rather than flavanones. These results did not totally agree with previous studies, which showed that the majority of citrus species largely accumulate flavanones and flavones (Tripoli et al., 2007; Wang et al., 2017b; Zhao et al., 2021). In addition, 123 DAFs were found in ct2 vs cj2, and the highest content of total flavonoids was 180DAF (**Figure 2A**). These 123 DAFs might be the flavonoids that cause the significant difference between ct2 and cj2. Heatmap analysis showed that the flavonoid content in ct was higher than cj (**Figure 3D**). Combined with DAFs analysis (**Figure 4**), rootstock grafting had the greatest impact on flavones, which might be because flavonoids were the most diverse flavonoid in sweet orange ‘Newhall’ peels. These flavonoids have the potential to generate anti-inflammatory, lipid and cholesterol-lowering, and other pharmacological actions that benefit human health (Kanaze et al., 2007; Li and Schluesener, 2017). KOG functional annotation found that many genes were significantly enriched in “Translation, ribosomal structure, and biogenesis”, “Transcription”, “Secondary metabolites biosynthesis, transport, and catabolism”, and “Signal transduction mechanisms” (**Figure 5B**), all of which were related to flavonoid biosynthesis (Liu et al., 2016; Wang et al., 2017a). GO and KEGG enrichment analysis found that many genes annotated to flavonoid pathway, and these genes might affect flavonoid biosynthesis. The flavonoid biosynthesis was tightly correlated with these biological functions (Jaakola, 2013; Li et al., 2020).

The flavonoid biosynthesis is determined by both structural and regulatory genes. Previous studies have demonstrated that many members of the *AP2/ERF-ERF* and *MYB* families play important roles in regulating flavonoid biosynthesis, such as *NtMYB12*, *PpMYB114*, *PpERF9*, *MsMYB62*, *FaMYB5*, *MdMYB9*, *CitERF32/33* (Song et al., 2019; Zhao et al., 2021; Jiang et al., 2023; Li et al., 2023; Meng et al., 2023; Ni et al., 2023; Yang et al., 2023). Surprisingly, two of the top three TF families were *AP2/ERF-ERF* family and *MYB* family, indicating their important functions in flavonoid biosynthesis (**Figure 9B**). Therefore, co-expressed was performed and found that *CHS* was positively correlated with one of the *AP2/ERF-ERF* members (*DREB3*). *CHS* and *CHI* were identified as rate-limiting genes, affecting downstream structural genes and flavonoid biosynthesis (Ngaki et al., 2012; Ban et al., 2018). Therefore, *DREB3* could activate the expression of *CHS* and thus regulate flavonoid biosynthesis. In addition, 18 TFs might be involved in the flavonoid biosynthesis in citrus peel after rootstock grafting, but further verification is needed. The regulatory mechanism of flavonoid biosynthesis is extremely complex. So far, only a few TFs have been identified to regulate flavonoid biosynthesis in citrus, and there are many more to be explored and verified in the future. Our study laid a foundation for the verification of the regulatory mechanism of flavonoids in citrus.

## Conclusion

**Figure 11:**
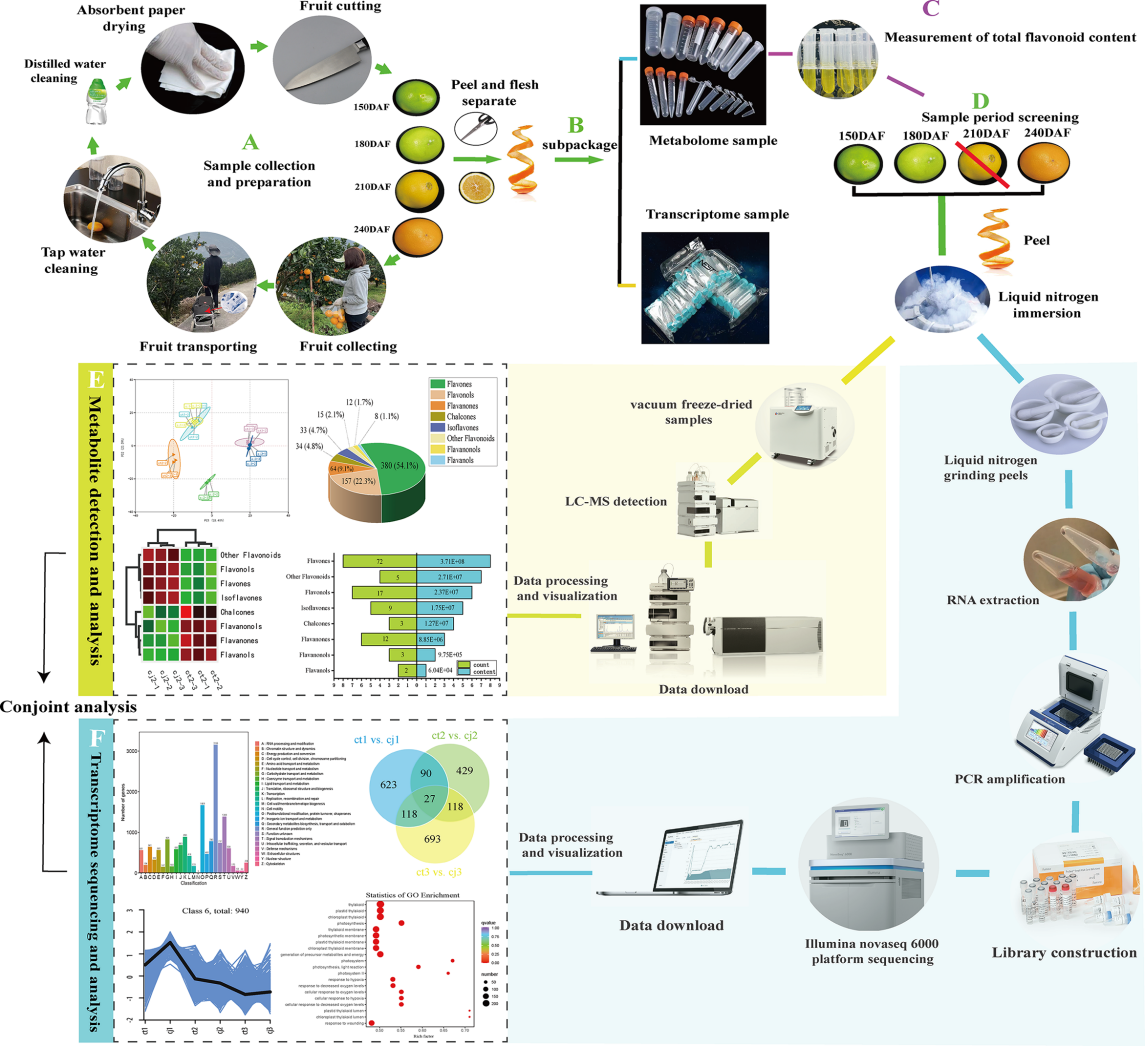
The flow chart of the whole experiment. The diagram mainly included the collection of samples, the processing of samples, the metabolome and transcriptome detection, and finally the conjoint analysis to reach the conclusion.

**Figure 12:**
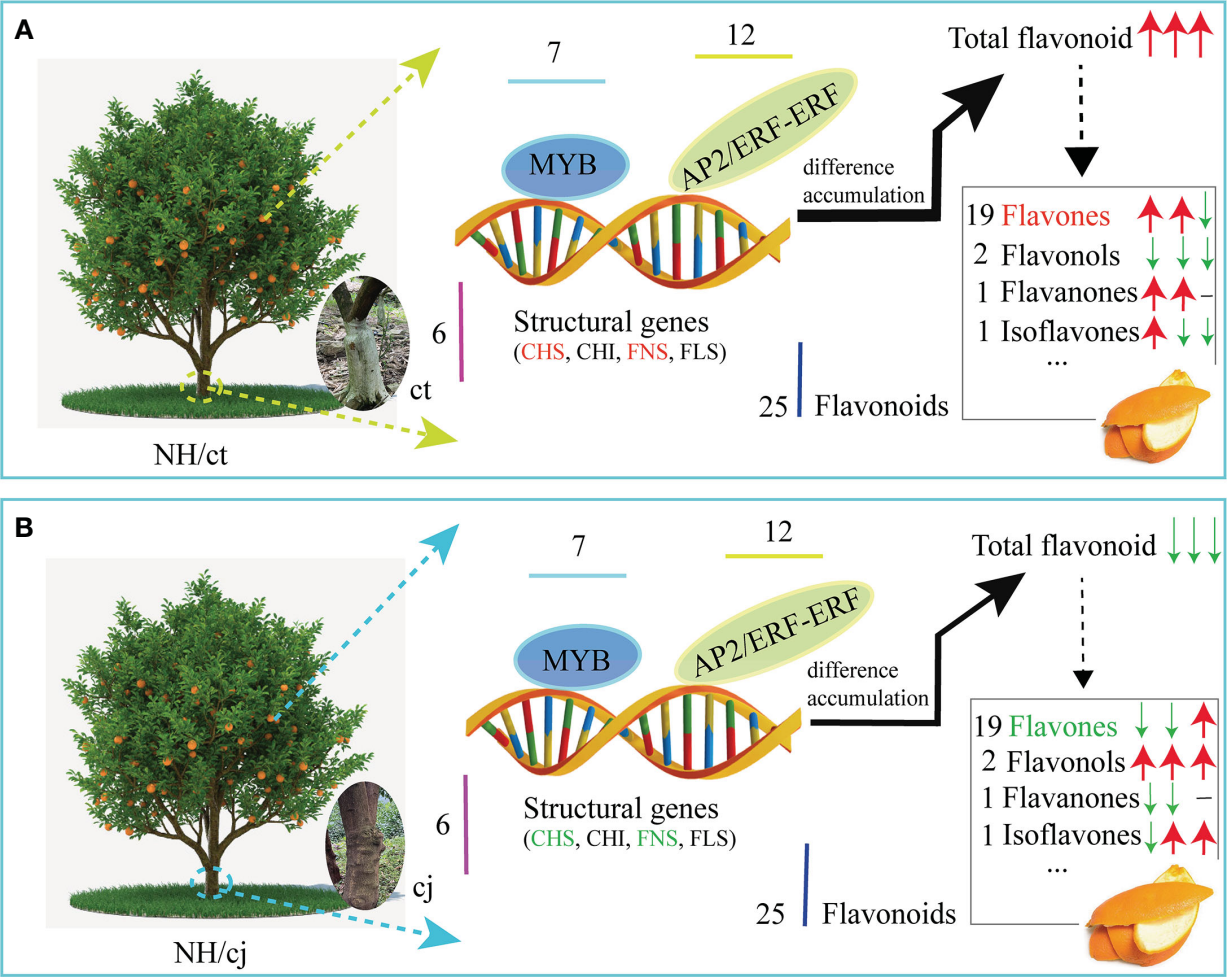
A hypothetical model for the involvement of flavonoids, structural genes and TFs in flavonoid biosynthesis under different rootstock grafting conditions in sweet orange ‘Newhall’ peels. The red arrows represented an increase, and the green arrows represented a decrease. The three arrows in sequence showed the variation trend in total flavonoids at 150DAF, 180DAF and 240DAF, respectively. The black horizontal lines indicated no detection.

Our study discovered 703 flavonoids in ct and cj peels during fruit expansion period, late fruit expansion period, and fruit ripening period. Twenty-one flavonoids were significantly affected by different developmental stages, and four flavonoids were significantly influenced with different rootstocks grafting. Flavones were the most affected by different rootstock grafting. Transcriptomic data revealed 38 candidate genes enriched in the flavonoid pathway. Based on the correlation analysis and gene co-expression network analysis, 6 unigenes for *CHS*, *CHI*, *FNS* and *FLS* and 19 hub TFs played an important role in regulating flavonoids biosynthesis. Our study revealed flavonoid compositions in ct and cj peels and elucidated the molecular regulation of flavonoid biosynthesis under various rootstock grafting circumstances (**Figures 11**, **12**). These findings would serve as a foundation for the further development and application of citrus peels. In addition, this research contributed to a better understanding of flavonoid biosynthesis pathway through differential rootstock grafting and provided the groundwork for future citrus molecular biology research and breeding.

## Data availability statement

The data presented in the study are deposited in the NCBI Sequence Read Archive Bioproject repository, accession number PRJNA953932.

## Author contributions

QL: Conceptualization, Data curation, Writing-original draft. JY, WZ and JW: Investigation, Validation. LL and GS: Data curation. XW, HD and MZ: Resources. ZW and BX: Funding acquisition, Resources, Project administration. All authors contributed to the article and approved the submitted version.
