# Supplementary material for: Hetero-grafting affects flavonoid biosynthesis in sweet orange 'Newhall' (Citrus sinensis) peels: a metabolomics and transcriptomics analysis
Source: Front Plant Sci. 2023 Jul 3;14:1218426. doi: 10.3389/fpls.2023.1218426 (PMC10351390; doi:10.3389/fpls.2023.1218426)
Supplement: Supplementary file 2 [file DataSheet_1.pdf]

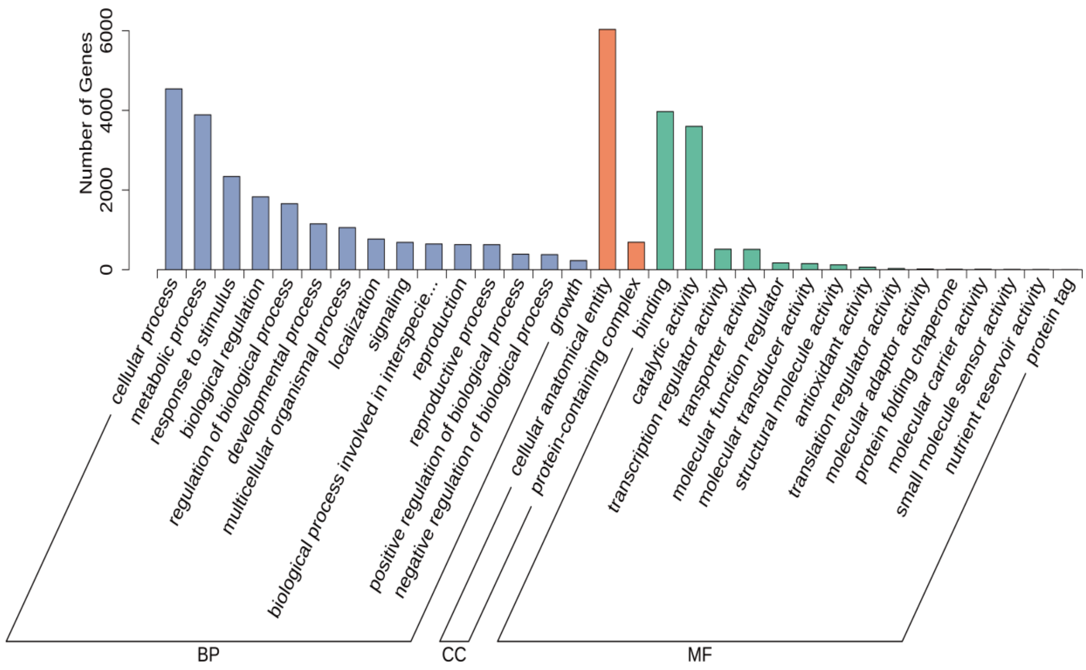

Fig. S1. GO classification histogram of DEGs.

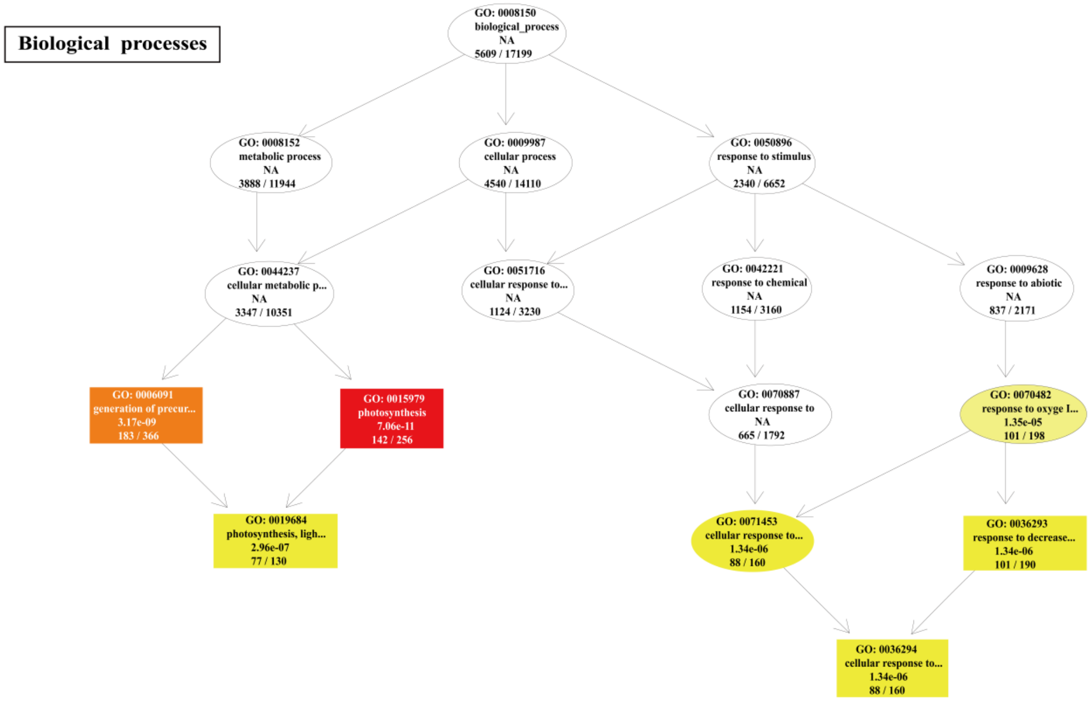

Fig. S2. Enriched directed acyclic graph (DAG) diagram of GO entries in Biological Processes. Each node represents a GO entry, the rectangle represents the top 5 GO entries selected, and the ellipse represents the included nodes. The colors of rectangles and ovals represent relative enrichment, ranging from bright yellow to dark red, with lower and lower P-values and higher and higher significance, while white represents insignificance. Each node displays 4 rows of data, respectively representing the ID, function, corrected P-value, the number of differential genes and the total number of genes of the GO entry.

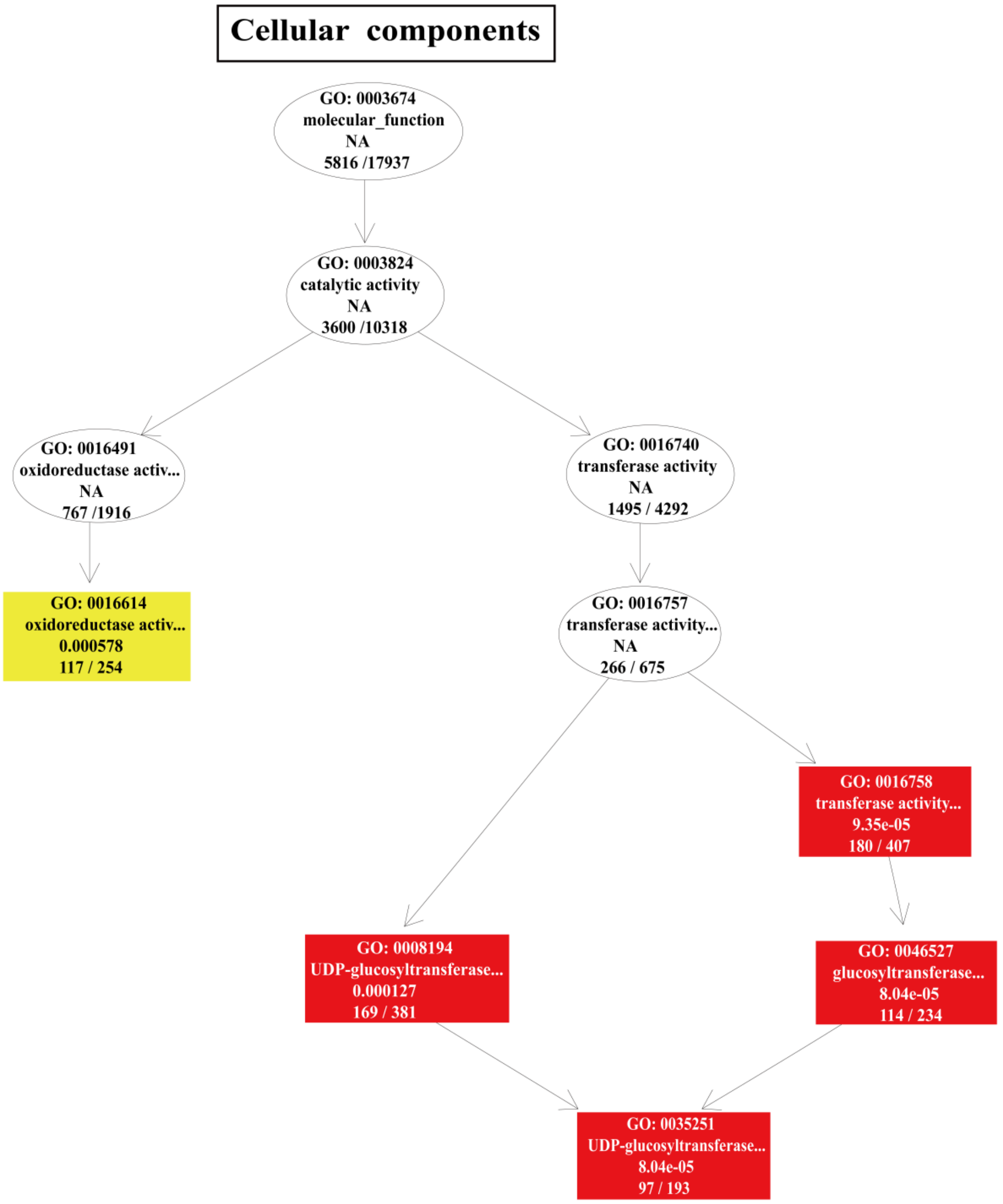

Fig. S3. Enriched directed acyclic graph (DAG) diagram of GO entries in Cellular Components. White represents no significance, yellow represents low significance, and red represents high significance.

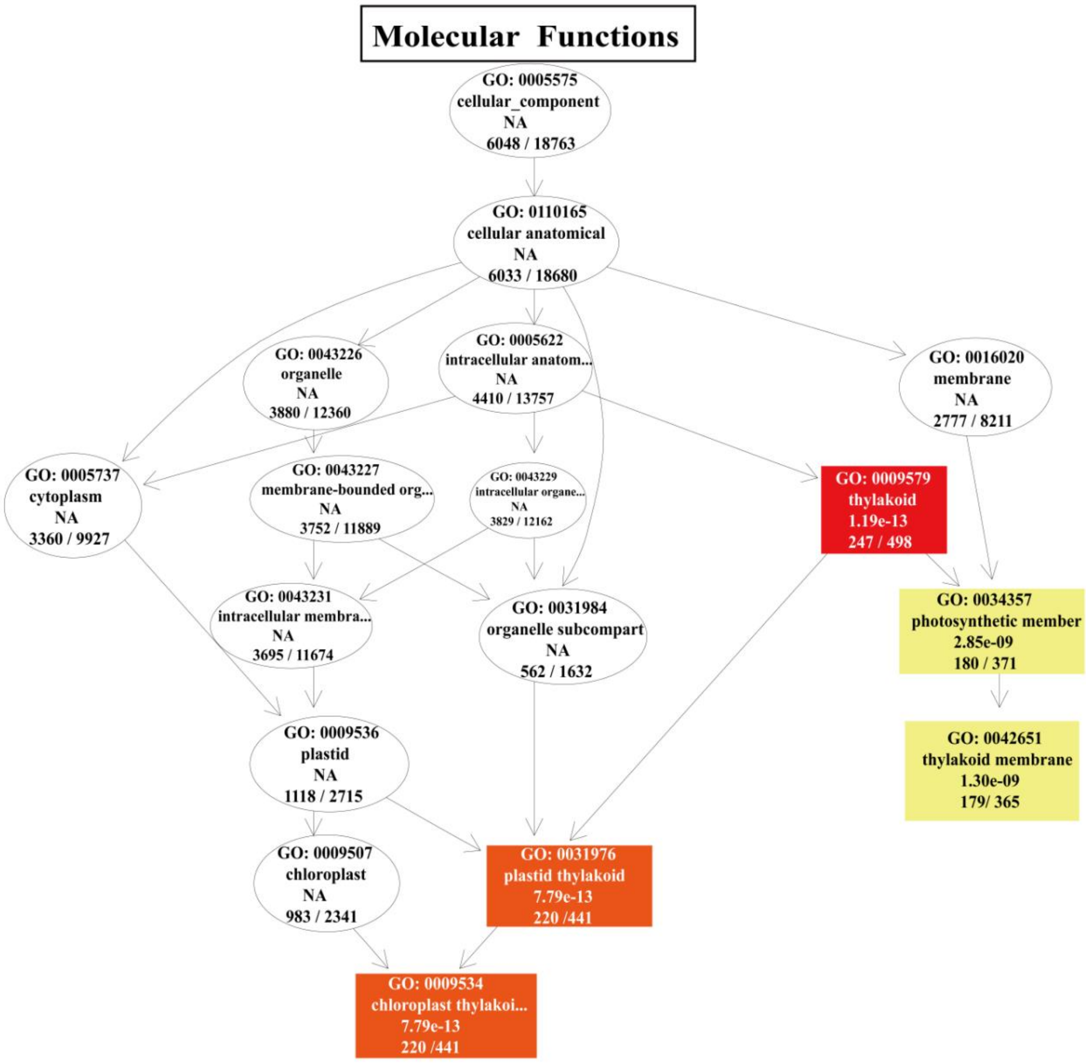

Fig. S4. Enriched directed acyclic graph (DAG) diagram of GO entries in Molecular Functions. The colors of rectangles and ovals represent relative enrichment, ranging from bright yellow to dark red, with lower and lower P-values and higher and higher significance, while white represents insignificance.
